# Editorial: Challenges and their implications for the clinical practice of head and neck cancer

**DOI:** 10.3389/fonc.2023.1131639

**Published:** 2023-01-18

**Authors:** Markus Wirth, Benedikt Schmidl, Barbara Wollenberg, Steffi Pigorsch

**Affiliations:** ^1^ Department of Otolaryngology Head and Neck Surgery, Klinikum rechts der Isar, Technical University Munich, Munich, Germany; ^2^ Department of RadioOncology and Radiotherapy, Klinikum rechts der Isar, Technical University Munich, Munich, Germany

**Keywords:** HNSCC, treatment related side effects, quality of life, surgery, radiotherapy - adverse effects

Head and neck squamous cell carcinoma (HNSCC) with its heterogeneous character, in part limited survival rates, and therapeutic options with significant side effects pose a serious challenge in clinical practice. A multidisciplinary and if needed multimodal approach including surgery, radio(chemo)therapy, and immuno- or chemotherapy is therefore the treatment of choice, with strategies changing and evolving constantly. In this special edition, latest advances in therapy, the quality of life and treatment-related side effects are highlighted.

In the clinical practice, the presentation with advanced clinical stages at the time of diagnosis is a defining feature for many head and neck cancers. Early diagnosis proved to be one of the most important prognostic factors, with late diagnosis leading to significantly impaired survival rates. The first section of this special edition therefore involves the advances in head and neck cancer diagnostics, including strategies for earlier diagnosis, but also prevention and prediction of patients most at risk. The articles range from nomograms predicting outcomes and important prognostic factors in laryngeal squamous cell carcinoma to saliva-based liquid biopsies, detecting tumor-derived components in a non-invasive way. In another attempt to improve the diagnostic options for head and neck cancer, diffusion-weighted MRI imaging is introduced to differentiate benign and malignant tumors of the parotid gland.

The second subject of this issue are advances in the treatment of head and neck cancer, with a special focus on the balance between the safe elimination of cancer, while also preserving functionality and the quality of life of patients. A selection of articles highlights the latest strategies in surgery. Based on the sonographic depth of tumor invasion, the stratified surgical dissection of the neck lymph nodes in tongue squamous cell carcinoma without apparent clinical lymph node metastasis is investigated. Another study sheds light on risk factors for postoperative complications in head and neck cancer patients after reconstruction with a free flap. Patients with certain preoperative conditions need to be screened adequately, to either monitor potential adverse effects more closely or stratify patients into different risk groups to design a more personalized treatment.

The third and last subject of this special edition are advances in chemo- and radiotherapy in HNSCC. There is a special focus on nasopharyngeal carcinoma, one of the most challenging entities of cancer of the head and neck, at the crossroads of surgery, radio- and chemotherapy. Intensity-modulated radiotherapy, prophylactic neck irradiation, and induction chemotherapy followed by concurrent chemotherapy are discussed. Furthermore, transcatheter arterial chemoembolization for oral cancer with the comorbidity of oral hemorrhage is introduced. Many therapies result in acceptable outcome, but patients are paying with side effects. One of the treatment modalities discussed in this special edition is cisplatin in advanced nasopharyngeal carcinoma, a highly effective chemotherapeutic agent, but at the cost of severe drug-induced adverse effects. The use of a lower dose of cisplatin, as demonstrated in a phase 2 trial in nasopharyngeal carcinoma, leads to similar outcomes, with a significantly lower number of side effects. In addition, the safety of intensity-modulated radiotherapy is demonstrated, while cerebrovascular disease after radiotherapy is another example of a potential side effect. Therefore, the patients need to be informed during the process of shared decision making on the indicated therapy. All these interacting factors in treating patients for cancer should be considered when selecting a treatment modality in clinical practice.

In summary, the articles in this special edition provide a comprehensive overview on the challenges in the daily treatment of patients with head and neck cancer. Surgery, radiotherapy, immunotherapy, and chemotherapy go hand in hand in the current oncological therapy. While radical therapeutic options were the treatment of choice in the past decades, the treatment strategies highlighted in this special edition display ways to preserve the patient’s quality of life and ensure a sufficient elimination of head and neck cancer.

## Author contributions

All authors listed have made a substantial, direct, and intellectual contribution to the work and approved it for publication.

